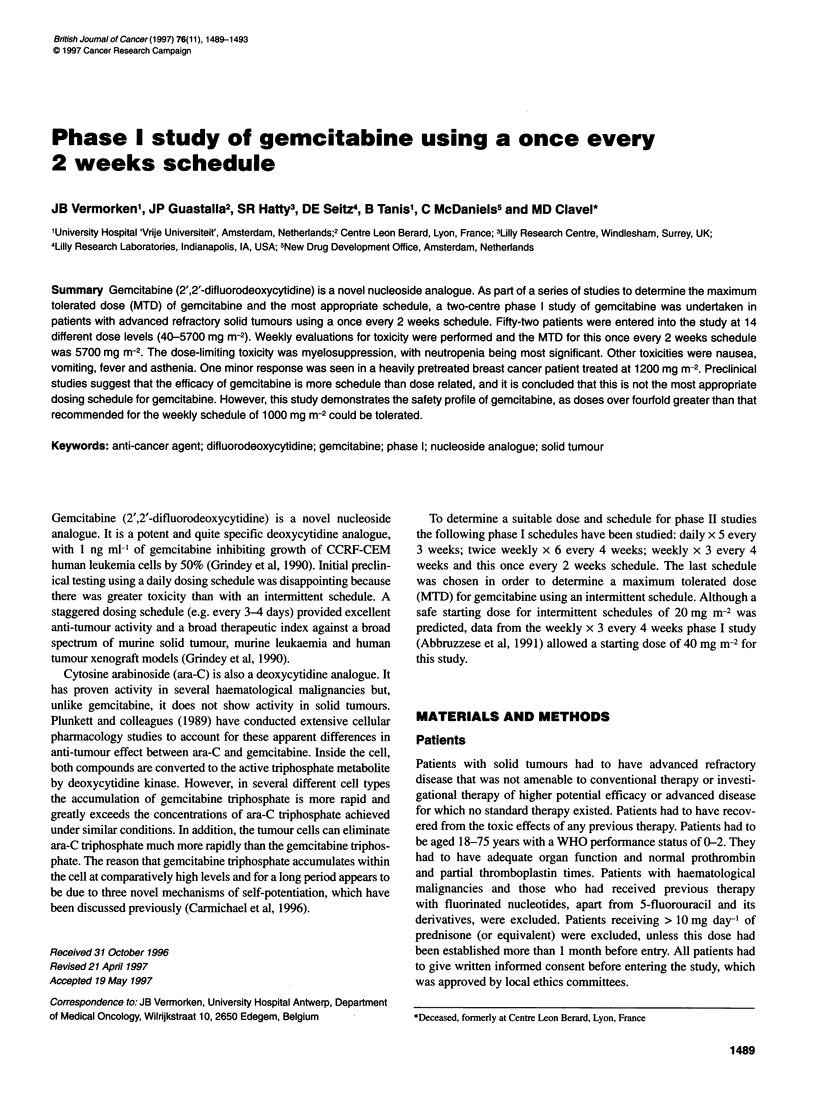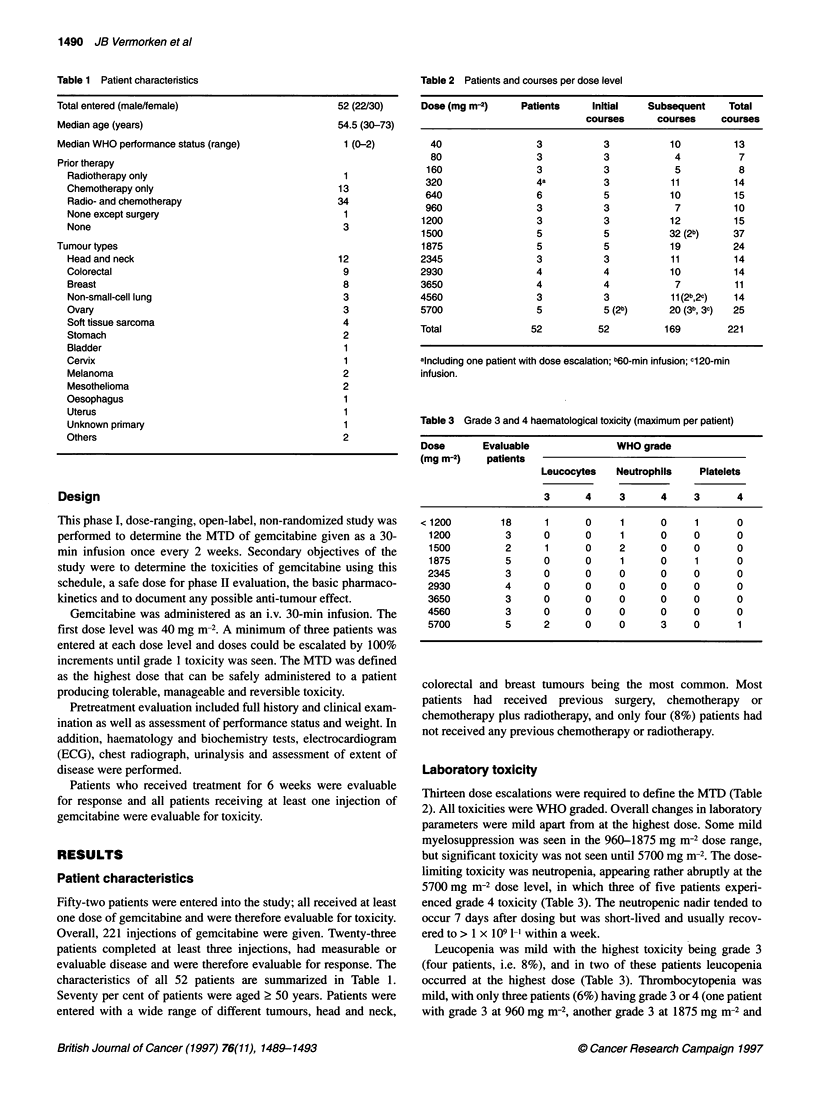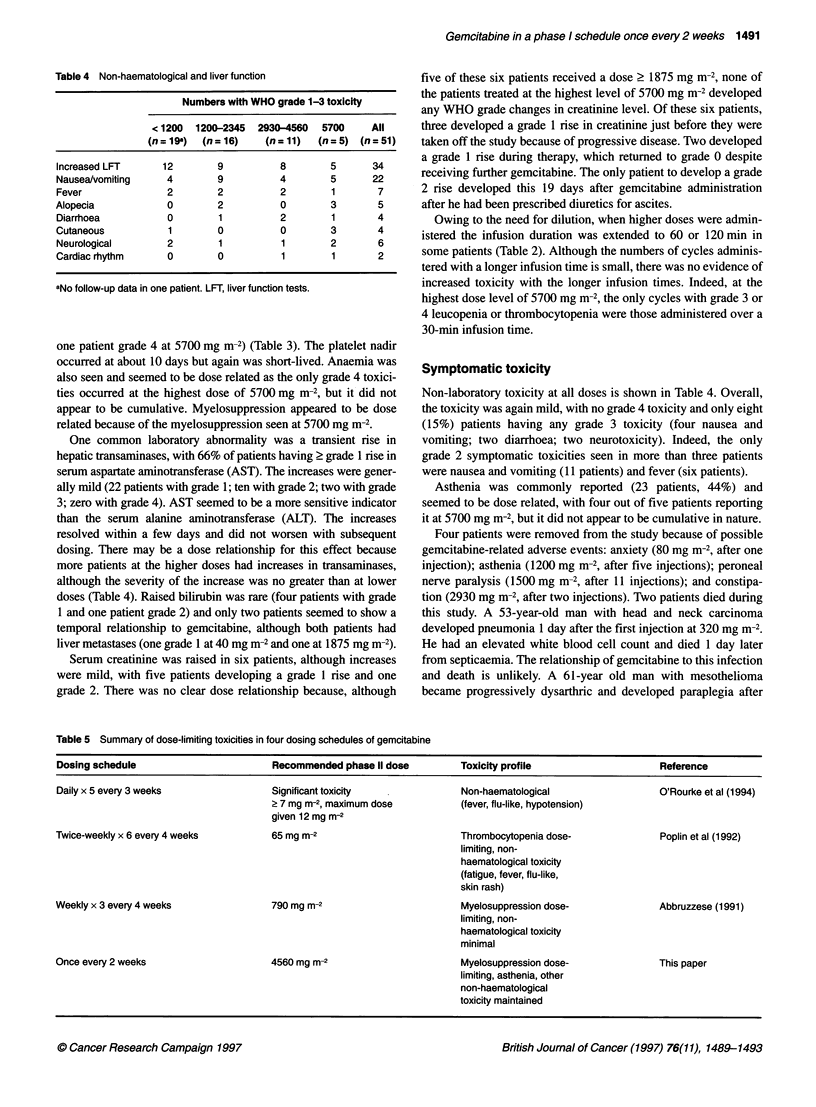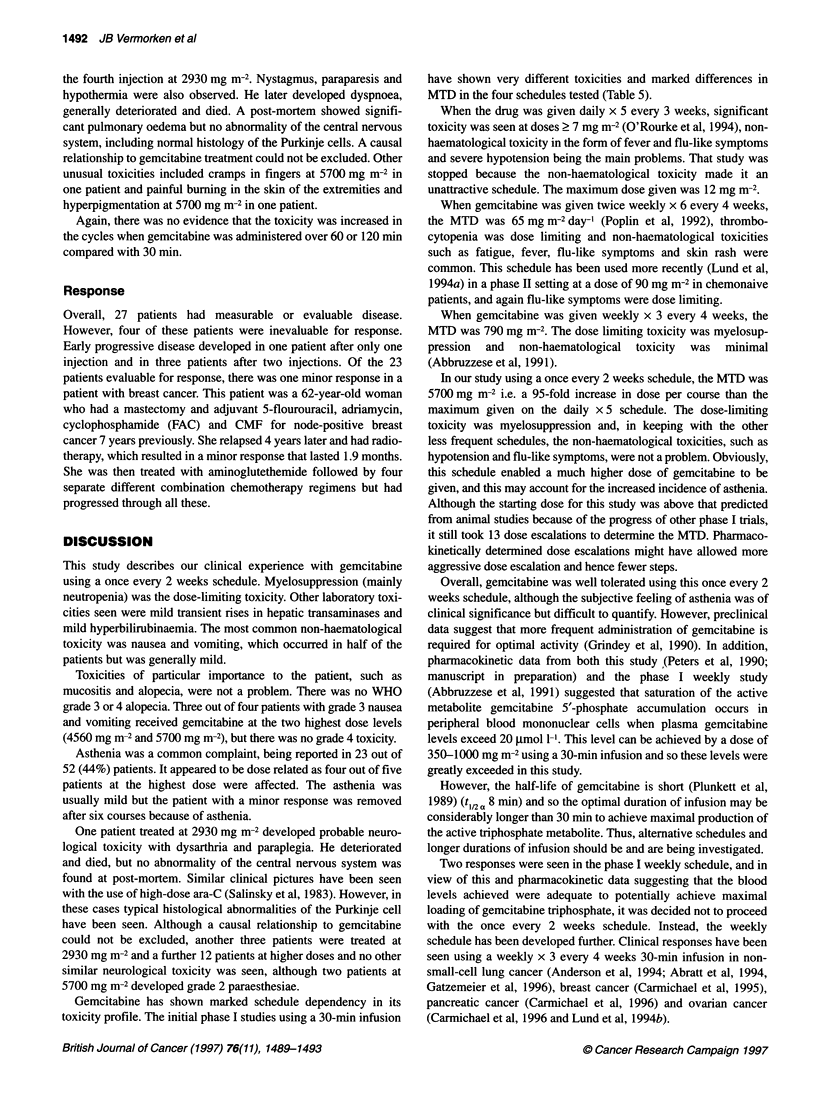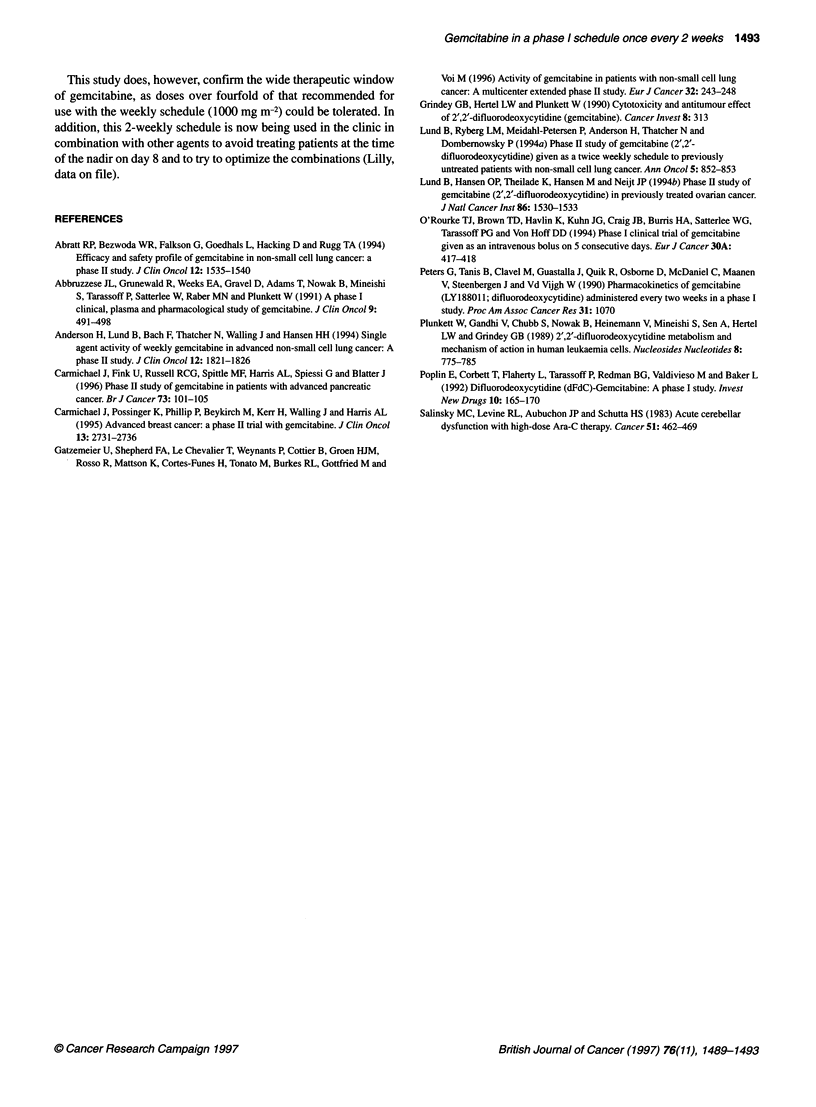# Phase I study of gemcitabine using a once every 2 weeks schedule.

**DOI:** 10.1038/bjc.1997.583

**Published:** 1997

**Authors:** J. B. Vermorken, J. P. Guastalla, S. R. Hatty, D. E. Seitz, B. Tanis, C. McDaniels, M. D. Clavel

**Affiliations:** University Hospital Vrije Universiteit, Amsterdam, Netherlands.

## Abstract

Gemcitabine (2',2'-difluorodeoxycytidine) is a novel nucleoside analogue. As part of a series of studies to determine the maximum tolerated dose (MTD) of gemcitabine and the most appropriate schedule, a two-centre phase I study of gemcitabine was undertaken in patients with advanced refractory solid tumours using a once every 2 weeks schedule. Fifty-two patients were entered into the study at 14 different dose levels (40-5700 mg m-2). Weekly evaluations for toxicity were performed and the MTD for this once every 2 weeks schedule was 5700 mg m-2. The dose-limiting toxicity was myelosuppression, with neutropenia being most significant. Other toxicities were nausea, vomiting, fever and asthenia. One minor response was seen in a heavily pretreated breast cancer patient treated at 1200 mg m-2. Preclinical studies suggest that the efficacy of gemcitabine is more schedule than dose related, and it is concluded that this is not the most appropriate dosing schedule for gemcitabine. However, this study demonstrates the safety profile of gemcitabine, as doses over fourfold greater than that recommended for the weekly schedule of 1000 mg m-2 could be tolerated.


British Journal of Cancer (1997) 76(11), 1489–1493
© 1997 Cancer Research Campaign

### Phase I study of gemcitabine using a once every 2 weeks schedule

**JB Vermorken[1], JP Guastalla[2], SR Hatty[3], DE Seitz[4], B Tanis[1], C McDaniels[5] and MD Clavel***

[1]University Hospital 'Vrije Universiteit', Amsterdam, Netherlands;[2] Centre Leon Berard, Lyon, France; [3]Lilly Research Centre, Windlesham, Surrey, UK; [4]Lilly Research Laboratories, Indianapolis, IA, USA; [5]New Drug Development Office, Amsterdam, Netherlands

**Summary** Gemcitabine (2′,2′-difluorodeoxycytidine) is a novel nucleoside analogue. As part of a series of studies to determine the maximum tolerated dose (MTD) of gemcitabine and the most appropriate schedule, a two-centre phase I study of gemcitabine was undertaken in patients with advanced refractory solid tumours using a once every 2 weeks schedule. Fifty-two patients were entered into the study at 14 different dose levels (40–5700 mg m$^{-2}$). Weekly evaluations for toxicity were performed and the MTD for this once every 2 weeks schedule was 5700 mg m$^{-2}$. The dose-limiting toxicity was myelosuppression, with neutropenia being most significant. Other toxicities were nausea, vomiting, fever and asthenia. One minor response was seen in a heavily pretreated breast cancer patient treated at 1200 mg m$^{-2}$. Preclinical studies suggest that the efficacy of gemcitabine is more schedule than dose related, and it is concluded that this is not the most appropriate dosing schedule for gemcitabine. However, this study demonstrates the safety profile of gemcitabine, as doses over fourfold greater than that recommended for the weekly schedule of 1000 mg m$^{-2}$ could be tolerated.

**Keywords:** anti-cancer agent; difluorodeoxycytidine; gemcitabine; phase I; nucleoside analogue; solid tumour

Gemcitabine (2′,2′-difluorodeoxycytidine) is a novel nucleoside analogue. It is a potent and quite specific deoxycytidine analogue, with 1 ng ml$^{-1}$ of gemcitabine inhibiting growth of CCRF-CEM human leukemia cells by 50% (Grindey et al, 1990). Initial preclinical testing using a daily dosing schedule was disappointing because there was greater toxicity than with an intermittent schedule. A staggered dosing schedule (e.g. every 3–4 days) provided excellent anti-tumour activity and a broad therapeutic index against a broad spectrum of murine solid tumour, murine leukaemia and human tumour xenograft models (Grindey et al, 1990).

Cytosine arabinoside (ara-C) is also a deoxycytidine analogue. It has proven activity in several haematological malignancies but, unlike gemcitabine, it does not show activity in solid tumours. Plunkett and colleagues (1989) have conducted extensive cellular pharmacology studies to account for these apparent differences in anti-tumour effect between ara-C and gemcitabine. Inside the cell, both compounds are converted to the active triphosphate metabolite by deoxycytidine kinase. However, in several different cell types the accumulation of gemcitabine triphosphate is more rapid and greatly exceeds the concentrations of ara-C triphosphate achieved under similar conditions. In addition, the tumour cells can eliminate ara-C triphosphate much more rapidly than the gemcitabine triphosphate. The reason that gemcitabine triphosphate accumulates within the cell at comparatively high levels and for a long period appears to be due to three novel mechanisms of self-potentiation, which have been discussed previously (Carmichael et al, 1996).

*Received 31 October 1996*
*Revised 21 April 1997*
*Accepted 19 May 1997*

*Correspondence to:* JB Vermorken, University Hospital Antwerp, Department of Medical Oncology, Wilrijkstraat 10, 2650 Edegem, Belgium

To determine a suitable dose and schedule for phase II studies the following phase I schedules have been studied: daily × 5 every 3 weeks; twice weekly × 6 every 4 weeks; weekly × 3 every 4 weeks and this once every 2 weeks schedule. The last schedule was chosen in order to determine a maximum tolerated dose (MTD) for gemcitabine using an intermittent schedule. Although a safe starting dose for intermittent schedules of 20 mg m$^{-2}$ was predicted, data from the weekly × 3 every 4 weeks phase I study (Abbruzzese et al, 1991) allowed a starting dose of 40 mg m$^{-2}$ for this study.

### MATERIALS AND METHODS

### Patients

Patients with solid tumours had to have advanced refractory disease that was not amenable to conventional therapy or investigational therapy of higher potential efficacy or advanced disease for which no standard therapy existed. Patients had to have recovered from the toxic effects of any previous therapy. Patients had to be aged 18–75 years with a WHO performance status of 0–2. They had to have adequate organ function and normal prothrombin and partial thromboplastin times. Patients with haematological malignancies and those who had received previous therapy with fluorinated nucleotides, apart from 5-fluorouracil and its derivatives, were excluded. Patients receiving > 10 mg day$^{-1}$ of prednisone (or equivalent) were excluded, unless this dose had been established more than 1 month before entry. All patients had to give written informed consent before entering the study, which was approved by local ethics committees.

*Deceased, formerly at Centre Leon Berard, Lyon, France

**Table 1** Patient characteristics

| | |
|---|---|
| Total entered (male/female) | 52 (22/30) |
| Median age (years) | 54.5 (30–73) |
| Median WHO performance status (range) | 1 (0–2) |
| Prior therapy | |
| Radiotherapy only | 1 |
| Chemotherapy only | 13 |
| Radio- and chemotherapy | 34 |
| None except surgery | 1 |
| None | 3 |
| Tumour types | |
| Head and neck | 12 |
| Colorectal | 9 |
| Breast | 8 |
| Non-small-cell lung | 3 |
| Ovary | 3 |
| Soft tissue sarcoma | 4 |
| Stomach | 2 |
| Bladder | 1 |
| Cervix | 1 |
| Melanoma | 2 |
| Mesothelioma | 2 |
| Oesophagus | 1 |
| Uterus | 1 |
| Unknown primary | 1 |
| Others | 2 |

**Table 2** Patients and courses per dose level

| Dose (mg m$^{-2}$) | Patients | Initial courses | Subsequent courses | Total courses |
|---|---|---|---|---|
| 40 | 3 | 3 | 10 | 13 |
| 80 | 3 | 3 | 4 | 7 |
| 160 | 3 | 3 | 5 | 8 |
| 320 | 4[a] | 3 | 11 | 14 |
| 640 | 6 | 5 | 10 | 15 |
| 960 | 3 | 3 | 7 | 10 |
| 1200 | 3 | 3 | 12 | 15 |
| 1500 | 5 | 5 | 32 (2[b]) | 37 |
| 1875 | 5 | 5 | 19 | 24 |
| 2345 | 3 | 3 | 11 | 14 |
| 2930 | 4 | 4 | 10 | 14 |
| 3650 | 4 | 4 | 7 | 11 |
| 4560 | 3 | 3 | 11 (2[b],2[c]) | 14 |
| 5700 | 5 | 5 (2[b]) | 20 (3[b], 3[c]) | 25 |
| Total | 52 | 52 | 169 | 221 |

[a]Including one patient with dose escalation; [b]60-min infusion; [c]120-min infusion.

**Table 3** Grade 3 and 4 haematological toxicity (maximum per patient)

| Dose (mg m$^{-2}$) | Evaluable patients | WHO grade | | | | | |
|---|---|---|---|---|---|---|---|
| | | Leucocytes | | Neutrophils | | Platelets | |
| | | 3 | 4 | 3 | 4 | 3 | 4 |
| < 1200 | 18 | 1 | 0 | 1 | 0 | 1 | 0 |
| 1200 | 3 | 0 | 0 | 1 | 0 | 0 | 0 |
| 1500 | 2 | 1 | 0 | 2 | 0 | 0 | 0 |
| 1875 | 5 | 0 | 0 | 1 | 0 | 1 | 0 |
| 2345 | 3 | 0 | 0 | 0 | 0 | 0 | 0 |
| 2930 | 4 | 0 | 0 | 0 | 0 | 0 | 0 |
| 3650 | 3 | 0 | 0 | 0 | 0 | 0 | 0 |
| 4560 | 3 | 0 | 0 | 0 | 0 | 0 | 0 |
| 5700 | 5 | 2 | 0 | 0 | 3 | 0 | 1 |

### Design

This phase I, dose-ranging, open-label, non-randomized study was performed to determine the MTD of gemcitabine given as a 30-min infusion once every 2 weeks. Secondary objectives of the study were to determine the toxicities of gemcitabine using this schedule, a safe dose for phase II evaluation, the basic pharmacokinetics and to document any possible anti-tumour effect.

Gemcitabine was administered as an i.v. 30-min infusion. The first dose level was 40 mg m$^{-2}$. A minimum of three patients was entered at each dose level and doses could be escalated by 100% increments until grade 1 toxicity was seen. The MTD was defined as the highest dose that can be safely administered to a patient producing tolerable, manageable and reversible toxicity.

Pretreatment evaluation included full history and clinical examination as well as assessment of performance status and weight. In addition, haematology and biochemistry tests, electrocardiogram (ECG), chest radiograph, urinalysis and assessment of extent of disease were performed.

Patients who received treatment for 6 weeks were evaluable for response and all patients receiving at least one injection of gemcitabine were evaluable for toxicity.

### RESULTS

### Patient characteristics

Fifty-two patients were entered into the study; all received at least one dose of gemcitabine and were therefore evaluable for toxicity. Overall, 221 injections of gemcitabine were given. Twenty-three patients completed at least three injections, had measurable or evaluable disease and were therefore evaluable for response. The characteristics of all 52 patients are summarized in Table 1. Seventy per cent of patients were aged ≥ 50 years. Patients were entered with a wide range of different tumours, head and neck, colorectal and breast tumours being the most common. Most patients had received previous surgery, chemotherapy or chemotherapy plus radiotherapy, and only four (8%) patients had not received any previous chemotherapy or radiotherapy.

### Laboratory toxicity

Thirteen dose escalations were required to define the MTD (Table 2). All toxicities were WHO graded. Overall changes in laboratory parameters were mild apart from at the highest dose. Some mild myelosuppression was seen in the 960–1875 mg m$^{-2}$ dose range, but significant toxicity was not seen until 5700 mg m$^{-2}$. The dose-limiting toxicity was neutropenia, appearing rather abruptly at the 5700 mg m$^{-2}$ dose level, in which three of five patients experienced grade 4 toxicity (Table 3). The neutropenic nadir tended to occur 7 days after dosing but was short-lived and usually recovered to $> 1 \times 10^9$ l$^{-1}$ within a week.

Leucopenia was mild with the highest toxicity being grade 3 (four patients, i.e. 8%), and in two of these patients leucopenia occurred at the highest dose (Table 3). Thrombocytopenia was mild, with only three patients (6%) having grade 3 or 4 (one patient with grade 3 at 960 mg m$^{-2}$, another grade 3 at 1875 mg m$^{-2}$ and

© Cancer Research Campaign 1997

**Table 4** Non-haematological and liver function

| | Numbers with WHO grade 1–3 toxicity | | | | |
|---|---|---|---|---|---|
| | < 1200 ($n$ = 19[a]) | 1200–2345 ($n$ = 16) | 2930–4560 ($n$ = 11) | 5700 ($n$ = 5) | All ($n$ = 51) |
| Increased LFT | 12 | 9 | 8 | 5 | 34 |
| Nausea/vomiting | 4 | 9 | 4 | 5 | 22 |
| Fever | 2 | 2 | 2 | 1 | 7 |
| Alopecia | 0 | 2 | 0 | 3 | 5 |
| Diarrhoea | 0 | 1 | 2 | 1 | 4 |
| Cutaneous | 1 | 0 | 0 | 3 | 4 |
| Neurological | 2 | 1 | 1 | 2 | 6 |
| Cardiac rhythm | 0 | 0 | 1 | 1 | 2 |

[a]No follow-up data in one patient. LFT, liver function tests.

one patient grade 4 at 5700 mg m$^{-2}$) (Table 3). The platelet nadir occurred at about 10 days but again was short-lived. Anaemia was also seen and seemed to be dose related as the only grade 4 toxicities occurred at the highest dose of 5700 mg m$^{-2}$, but it did not appear to be cumulative. Myelosuppression appeared to be dose related because of the myelosuppression seen at 5700 mg m$^{-2}$.

One common laboratory abnormality was a transient rise in hepatic transaminases, with 66% of patients having ≥ grade 1 rise in serum aspartate aminotransferase (AST). The increases were generally mild (22 patients with grade 1; ten with grade 2; two with grade 3; zero with grade 4). AST seemed to be a more sensitive indicator than the serum alanine aminotransferase (ALT). The increases resolved within a few days and did not worsen with subsequent dosing. There may be a dose relationship for this effect because more patients at the higher doses had increases in transaminases, although the severity of the increase was no greater than at lower doses (Table 4). Raised bilirubin was rare (four patients with grade 1 and one patient grade 2) and only two patients seemed to show a temporal relationship to gemcitabine, although both patients had liver metastases (one grade 1 at 40 mg m$^{-2}$ and one at 1875 mg m$^{-2}$).

Serum creatinine was raised in six patients, although increases were mild, with five patients developing a grade 1 rise and one grade 2. There was no clear dose relationship because, although five of these six patients received a dose ≥ 1875 mg m$^{-2}$, none of the patients treated at the highest level of 5700 mg m$^{-2}$ developed any WHO grade changes in creatinine level. Of these six patients, three developed a grade 1 rise in creatinine just before they were taken off the study because of progressive disease. Two developed a grade 1 rise during therapy, which returned to grade 0 despite receiving further gemcitabine. The only patient to develop a grade 2 rise developed this 19 days after gemcitabine administration after he had been prescribed diuretics for ascites.

Owing to the need for dilution, when higher doses were administered the infusion duration was extended to 60 or 120 min in some patients (Table 2). Although the numbers of cycles administered with a longer infusion time is small, there was no evidence of increased toxicity with the longer infusion times. Indeed, at the highest dose level of 5700 mg m$^{-2}$, the only cycles with grade 3 or 4 leucopenia or thrombocytopenia were those administered over a 30-min infusion time.

### Symptomatic toxicity

Non-laboratory toxicity at all doses is shown in Table 4. Overall, the toxicity was again mild, with no grade 4 toxicity and only eight (15%) patients having any grade 3 toxicity (four nausea and vomiting; two diarrhoea; two neurotoxicity). Indeed, the only grade 2 symptomatic toxicities seen in more than three patients were nausea and vomiting (11 patients) and fever (six patients).

Asthenia was commonly reported (23 patients, 44%) and seemed to be dose related, with four out of five patients reporting it at 5700 mg m$^{-2}$, but it did not appear to be cumulative in nature.

Four patients were removed from the study because of possible gemcitabine-related adverse events: anxiety (80 mg m$^{-2}$, after one injection); asthenia (1200 mg m$^{-2}$, after five injections); peroneal nerve paralysis (1500 mg m$^{-2}$, after 11 injections); and constipation (2930 mg m$^{-2}$, after two injections). Two patients died during this study. A 53-year-old man with head and neck carcinoma developed pneumonia 1 day after the first injection at 320 mg m$^{-2}$. He had an elevated white blood cell count and died 1 day later from septicaemia. The relationship of gemcitabine to this infection and death is unlikely. A 61-year old man with mesothelioma became progressively dysarthric and developed paraplegia after

**Table 5** Summary of dose-limiting toxicities in four dosing schedules of gemcitabine

| Dosing schedule | Recommended phase II dose | Toxicity profile | Reference |
|---|---|---|---|
| Daily × 5 every 3 weeks | Significant toxicity ≥ 7 mg m$^{-2}$, maximum dose given 12 mg m$^{-2}$ | Non-haematological (fever, flu-like, hypotension) | O'Rourke et al (1994) |
| Twice-weekly × 6 every 4 weeks | 65 mg m$^{-2}$ | Thrombocytopenia dose-limiting, non-haematological toxicity (fatigue, fever, flu-like, skin rash) | Poplin et al (1992) |
| Weekly × 3 every 4 weeks | 790 mg m$^{-2}$ | Myelosuppression dose-limiting, non-haematological toxicity minimal | Abbruzzese (1991) |
| Once every 2 weeks | 4560 mg m$^{-2}$ | Myelosuppression dose-limiting, asthenia, other non-haematological toxicity maintained | This paper |

© Cancer Research Campaign 1997 

the fourth injection at 2930 mg $m^{-2}$. Nystagmus, paraparesis and hypothermia were also observed. He later developed dyspnoea, generally deteriorated and died. A post-mortem showed significant pulmonary oedema but no abnormality of the central nervous system, including normal histology of the Purkinje cells. A causal relationship to gemcitabine treatment could not be excluded. Other unusual toxicities included cramps in fingers at 5700 mg $m^{-2}$ in one patient and painful burning in the skin of the extremities and hyperpigmentation at 5700 mg $m^{-2}$ in one patient.

Again, there was no evidence that the toxicity was increased in the cycles when gemcitabine was administered over 60 or 120 min compared with 30 min.

### Response

Overall, 27 patients had measurable or evaluable disease. However, four of these patients were inevaluable for response. Early progressive disease developed in one patient after only one injection and in three patients after two injections. Of the 23 patients evaluable for response, there was one minor response in a patient with breast cancer. This patient was a 62-year-old woman who had a mastectomy and adjuvant 5-flourouracil, adriamycin, cyclophosphamide (FAC) and CMF for node-positive breast cancer 7 years previously. She relapsed 4 years later and had radiotherapy, which resulted in a minor response that lasted 1.9 months. She was then treated with aminoglutethemide followed by four separate different combination chemotherapy regimens but had progressed through all these.

### DISCUSSION

This study describes our clinical experience with gemcitabine using a once every 2 weeks schedule. Myelosuppression (mainly neutropenia) was the dose-limiting toxicity. Other laboratory toxicities seen were mild transient rises in hepatic transaminases and mild hyperbilirubinaemia. The most common non-haematological toxicity was nausea and vomiting, which occurred in half of the patients but was generally mild.

Toxicities of particular importance to the patient, such as mucositis and alopecia, were not a problem. There was no WHO grade 3 or 4 alopecia. Three out of four patients with grade 3 nausea and vomiting received gemcitabine at the two highest dose levels (4560 mg $m^{-2}$ and 5700 mg $m^{-2}$), but there was no grade 4 toxicity.

Asthenia was a common complaint, being reported in 23 out of 52 (44%) patients. It appeared to be dose related as four out of five patients at the highest dose were affected. The asthenia was usually mild but the patient with a minor response was removed after six courses because of asthenia.

One patient treated at 2930 mg $m^{-2}$ developed probable neurological toxicity with dysarthria and paraplegia. He deteriorated and died, but no abnormality of the central nervous system was found at post-mortem. Similar clinical pictures have been seen with the use of high-dose ara-C (Salinsky et al, 1983). However, in these cases typical histological abnormalities of the Purkinje cell have been seen. Although a causal relationship to gemcitabine could not be excluded, another three patients were treated at 2930 mg $m^{-2}$ and a further 12 patients at higher doses and no other similar neurological toxicity was seen, although two patients at 5700 mg $m^{-2}$ developed grade 2 paraesthesiae.

Gemcitabine has shown marked schedule dependency in its toxicity profile. The initial phase I studies using a 30-min infusion have shown very different toxicities and marked differences in MTD in the four schedules tested (Table 5).

When the drug was given daily × 5 every 3 weeks, significant toxicity was seen at doses ≥ 7 mg $m^{-2}$ (O'Rourke et al, 1994), non-haematological toxicity in the form of fever and flu-like symptoms and severe hypotension being the main problems. That study was stopped because the non-haematological toxicity made it an unattractive schedule. The maximum dose given was 12 mg $m^{-2}$.

When gemcitabine was given twice weekly × 6 every 4 weeks, the MTD was 65 mg $m^{-2}$ $day^{-1}$ (Poplin et al, 1992), thrombocytopenia was dose limiting and non-haematological toxicities such as fatigue, fever, flu-like symptoms and skin rash were common. This schedule has been used more recently (Lund et al, 1994*a*) in a phase II setting at a dose of 90 mg $m^{-2}$ in chemonaive patients, and again flu-like symptoms were dose limiting.

When gemcitabine was given weekly × 3 every 4 weeks, the MTD was 790 mg $m^{-2}$. The dose limiting toxicity was myelosuppression and non-haematological toxicity was minimal (Abbruzzese et al, 1991).

In our study using a once every 2 weeks schedule, the MTD was 5700 mg $m^{-2}$ i.e. a 95-fold increase in dose per course than the maximum given on the daily × 5 schedule. The dose-limiting toxicity was myelosuppression and, in keeping with the other less frequent schedules, the non-haematological toxicities, such as hypotension and flu-like symptoms, were not a problem. Obviously, this schedule enabled a much higher dose of gemcitabine to be given, and this may account for the increased incidence of asthenia. Although the starting dose for this study was above that predicted from animal studies because of the progress of other phase I trials, it still took 13 dose escalations to determine the MTD. Pharmacokinetically determined dose escalations might have allowed more aggressive dose escalation and hence fewer steps.

Overall, gemcitabine was well tolerated using this once every 2 weeks schedule, although the subjective feeling of asthenia was of clinical significance but difficult to quantify. However, preclinical data suggest that more frequent administration of gemcitabine is required for optimal activity (Grindey et al, 1990). In addition, pharmacokinetic data from both this study (Peters et al, 1990; manuscript in preparation) and the phase I weekly study (Abbruzzese et al, 1991) suggested that saturation of the active metabolite gemcitabine 5′-phosphate accumulation occurs in peripheral blood mononuclear cells when plasma gemcitabine levels exceed 20 μmol $l^{-1}$. This level can be achieved by a dose of 350–1000 mg $m^{-2}$ using a 30-min infusion and so these levels were greatly exceeded in this study.

However, the half-life of gemcitabine is short (Plunkett et al, 1989) ($t_{1/2\alpha}$ 8 min) and so the optimal duration of infusion may be considerably longer than 30 min to achieve maximal production of the active triphosphate metabolite. Thus, alternative schedules and longer durations of infusion should be and are being investigated.

Two responses were seen in the phase I weekly schedule, and in view of this and pharmacokinetic data suggesting that the blood levels achieved were adequate to potentially achieve maximal loading of gemcitabine triphosphate, it was decided not to proceed with the once every 2 weeks schedule. Instead, the weekly schedule has been developed further. Clinical responses have been seen using a weekly × 3 every 4 weeks 30-min infusion in non-small-cell lung cancer (Anderson et al, 1994; Abratt et al, 1994, Gatzemeier et al, 1996), breast cancer (Carmichael et al, 1995), pancreatic cancer (Carmichael et al, 1996) and ovarian cancer (Carmichael et al, 1996 and Lund et al, 1994*b*).

 © Cancer Research Campaign 1997

This study does, however, confirm the wide therapeutic window of gemcitabine, as doses over fourfold of that recommended for use with the weekly schedule (1000 mg $m^{-2}$) could be tolerated. In addition, this 2-weekly schedule is now being used in the clinic in combination with other agents to avoid treating patients at the time of the nadir on day 8 and to try to optimize the combinations (Lilly, data on file).

### REFERENCES

Abratt RP, Bezwoda WR, Falkson G, Goedhals L, Hacking D and Rugg TA (1994) Efficacy and safety profile of gemcitabine in non-small cell lung cancer: a phase II study. *J Clin Oncol* **12:** 1535–1540

Abbruzzese JL, Grunewald R, Weeks EA, Gravel D, Adams T, Nowak B, Mineishi S, Tarassoff P, Satterlee W, Raber MN and Plunkett W (1991) A phase I clinical, plasma and pharmacological study of gemcitabine. *J Clin Oncol* **9:** 491–498

Anderson H, Lund B, Bach F, Thatcher N, Walling J and Hansen HH (1994) Single agent activity of weekly gemcitabine in advanced non-small cell lung cancer: A phase II study. *J Clin Oncol* **12:** 1821–1826

Carmichael J, Fink U, Russell RCG, Spittle MF, Harris AL, Spiessi G and Blatter J (1996) Phase II study of gemcitabine in patients with advanced pancreatic cancer. *Br J Cancer* **73:** 101–105

Carmichael J, Possinger K, Phillip P, Beykirch M, Kerr H, Walling J and Harris AL (1995) Advanced breast cancer: a phase II trial with gemcitabine. *J Clin Oncol* **13:** 2731–2736

Gatzemeier U, Shepherd FA, Le Chevalier T, Weynants P, Cottier B, Groen HJM, Rosso R, Mattson K, Cortes-Funes H, Tonato M, Burkes RL, Gottfried M and Voi M (1996) Activity of gemcitabine in patients with non-small cell lung cancer: A multicenter extended phase II study. *Eur J Cancer* **32:** 243–248

Grindey GB, Hertel LW and Plunkett W (1990) Cytotoxicity and antitumour effect of 2′,2′-difluorodeoxycytidine (gemcitabine). *Cancer Invest* **8:** 313

Lund B, Ryberg LM, Meidahl-Petersen P, Anderson H, Thatcher N and Dombernowsky P (1994*a*) Phase II study of gemcitabine (2′,2′-difluorodeoxycytidine) given as a twice weekly schedule to previously untreated patients with non-small cell lung cancer. *Ann Oncol* **5:** 852–853

Lund B, Hansen OP, Theilade K, Hansen M and Neijt JP (1994*b*) Phase II study of gemcitabine (2′,2′-difluorodeoxycytidine) in previously treated ovarian cancer. *J Natl Cancer Inst* **86:** 1530–1533

O'Rourke TJ, Brown TD, Havlin K, Kuhn JG, Craig JB, Burris HA, Satterlee WG, Tarassoff PG and Von Hoff DD (1994) Phase I clinical trial of gemcitabine given as an intravenous bolus on 5 consecutive days. *Eur J Cancer* **30A:** 417–418

Peters G, Tanis B, Clavel M, Guastalla J, Quik R, Osborne D, McDaniel C, Maanen V, Steenbergen J and Vd Vijgh W (1990) Pharmacokinetics of gemcitabine (LY188011; difluorodeoxycytidine) administered every two weeks in a phase I study. *Proc Am Assoc Cancer Res* **31:** 1070

Plunkett W, Gandhi V, Chubb S, Nowak B, Heinemann V, Mineishi S, Sen A, Hertel LW and Grindey GB (1989) 2′,2′-difluorodeoxycytidine metabolism and mechanism of action in human leukaemia cells. *Nucleosides Nucleotides* **8:** 775–785

Poplin E, Corbett T, Flaherty L, Tarassoff P, Redman BG, Valdivieso M and Baker L (1992) Difluorodeoxycytidine (dFdC)-Gemcitabine: A phase I study. *Invest New Drugs* **10:** 165–170

Salinsky MC, Levine RL, Aubuchon JP and Schutta HS (1983) Acute cerebellar dysfunction with high-dose Ara-C therapy. *Cancer* **51:** 462–469

© Cancer Research Campaign 1997